# Persistent inner tepals and wings protect developing seeds of *Rheum nanum* from insect herbivory in Central Asian cold deserts

**DOI:** 10.1002/ece3.70179

**Published:** 2024-08-15

**Authors:** Yuting Li, Jannathan Mamut, Kaiqing Xie, Jing Zhao, Dunyan Tan

**Affiliations:** ^1^ Xinjiang Key Laboratory for Ecological Adaptation and Evolution of Extreme Environment Biology, College of Life Sciences Xinjiang Agricultural University Urumqi China; ^2^ Key Laboratory of Ministry of Education for Western Arid Region Grassland Resources and Ecology, College of Grassland Sciences Xinjiang Agricultural University Urumqi China

**Keywords:** desert plant, fruit wing, mechanical defense, persistent inner tepal, *Rheum nanum*, seed development

## Abstract

Although the postdispersal functions of diaspore (fruit and its appendages) have been reported, little is known about their protective/defensive functions. In this context, diaspores with appendages (persistent inner tepals and/or fruit wings) that experienced predispersal herbivory by insects in natural populations of *Rheum nanum* were investigated, and the seed abortion percentage, seed and embryo masses, and germination of seeds from diaspores with different categories of insect herbivory were measured and compared. Predispersal insect herbivory of *R*. *nanum* diaspores was prevalent in the four investigated populations, but the percentage of diaspores with appendages (persistent inner tepals and and/or fruit wings) damaged by insects was significantly higher than that of diaspores with the pericarp damaged by insects. Seeds from diaspores with gnawed appendages experienced significantly less damage than those with gnawed pericarps. Importantly, we conclude that fruit appendages of *R*. *nanum* help to mechanically protect developing seeds from predispersal insect herbivory.

## Graphical abstract

Although the postdispersal function of fruit appendages has been reported, little is known about their protective/defensive functions. In this context, diaspores with appendages (persistent inner tepals and/or fruit wings) that experienced predispersal herbivory by insects in natural populations of *Rheum nanum* were investigated. The seed abortion percentage, seed and embryo masses, and germination of seeds from diaspores with different categories of insect herbivory were measured and compared. Predispersal insect herbivory of *R*. *nanum* diaspores was prevalent in the four natural populations, but the percentage of diaspores with appendages (persistent inner tepals and and/or fruit wings) damaged by insects was significantly higher than that of diaspores with the pericarp damaged by insects. Seeds from diaspores with gnawed appendages experienced significantly less damage than those with gnawed pericarps. Importantly, we conclude that fruit appendages of *R. nanum* help to mechanically protect developing seeds from predispersal insect herbivory.

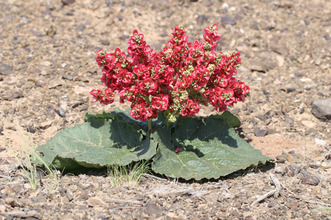

## 1 INTRODUCTION

Animal herbivory impacts the diversity and distribution of plant species (Gagic et al., 2016; Moles et al., 2003). Therefore, plants have evolved various defense mechanisms (e.g., constitutive or induced defenses) (Hunziker et al., 2021; Mitchell et al., 2016; Tortorici et al., 2022). Constitutive defenses can be either mechanical or chemical, and they can act independently or together (Bar & Shtein, 2019; Bonaventure et al., 2011; Hanley et al., 2007).

Mechanical defense is achieved via accessory structures such as spines, thorns, awns, and trichomes (Bobrov & Romanov, 2019; Grime et al., 1981). For example, the bright colorful thorns and spines of some species can serve as aposematic coloration to deter mammal herbivores (Lev‐Yadun, 2001, 2003), and spine length is related to amount of herbivory (Young et al., 2003). Furthermore, trichomes also can protect plants by decreasing insect oviposition (Handley et al., 2005), preventing larval movement (Figueiredo et al., 2013; Verheggen et al., 2009), reducing larval feeding (Dalin & Björkman, 2003; Kariyat et al., 2017), entrapping insects (Xing et al., 2017), harming the mouth and intestines of herbivores (Kariyat et al., 2017), or puncturing/injuring some insects directly (Quiring et al., 1992).

Fruit is not only the reproductive organs of angiosperms but also the important component, and some animals use the nutrient‐rich pre‐ and/or postdispersal fruits of angiosperms as food. Thus, fruit appendages are functionally important, and various studies have demonstrated their effects not only on facilitating seed dispersal by animals (Johnson et al., 2020; Ma et al., 2010), wind (Lu et al., 2013; Ma et al., 2010), or water (Mandák & Pyšek, 2001; Säumel & Kowarik, 2013) but also their role in promoting (Peart, 1981; Schöning et al., 2004) or inhibiting (Bhatt et al., 2017, 2019; El‐Keblawy et al., 2014; Pan et al., 2021, Zhu et al., 2022) seed germination. However, little is known about the antiherbivore function of fruit appendages during fruit development.

*Rheum nanum* Siev. ex Pall. (Polygonaceae) is a perennial ephemeral herb that occurs in the cold desert region of Central Asia at altitudes of 700–2000 m a.s.l. (Bao & Grabovskaya‐Borodina, 2003). This species begins to flower in mid‐April, and achenes mature in early June. During the period of achene development, the succulent fruit has three red‐ribbed wings, and it is always covered by green persistent inner tepals. Therefore, the diaspores of this species include a winged‐achene, persistent inner tepals, and a triangular seed composed of a white embryo and endosperm. Our field observations revealed that each plant of *R*. *nanum* can produce more than 500 diaspores in the four investigated populations of the study area, and the insect herbivory to diaspores is very common before dispersal. Both persistent inner tepals and winged achene of *R*. *nanum* experience predispersal insect herbivory in natural populations, and the persistent inner tepals and achene wings experienced heavier insect damage than the pericarp. Thus, we hypothesized that persistent inner tepals and fruit wings of *R*. *nanum* can mechanically protect the developing seeds from damage by insect herbivores.

To test this hypothesis, we addressed the following questions: (1) How prevalent is predispersal insect herbivory in natural populations of *R*. *nanum*? (2) Do seeds abort when diaspores receive predispersal insect herbivory? and (3) What are the impacts of predispersal insect herbivory on seed mass and germination of *R*. *nanum*?

## 2 MATERIALS AND METHODS

### 2.1 Description of study site and material

**FIGURE 1:**
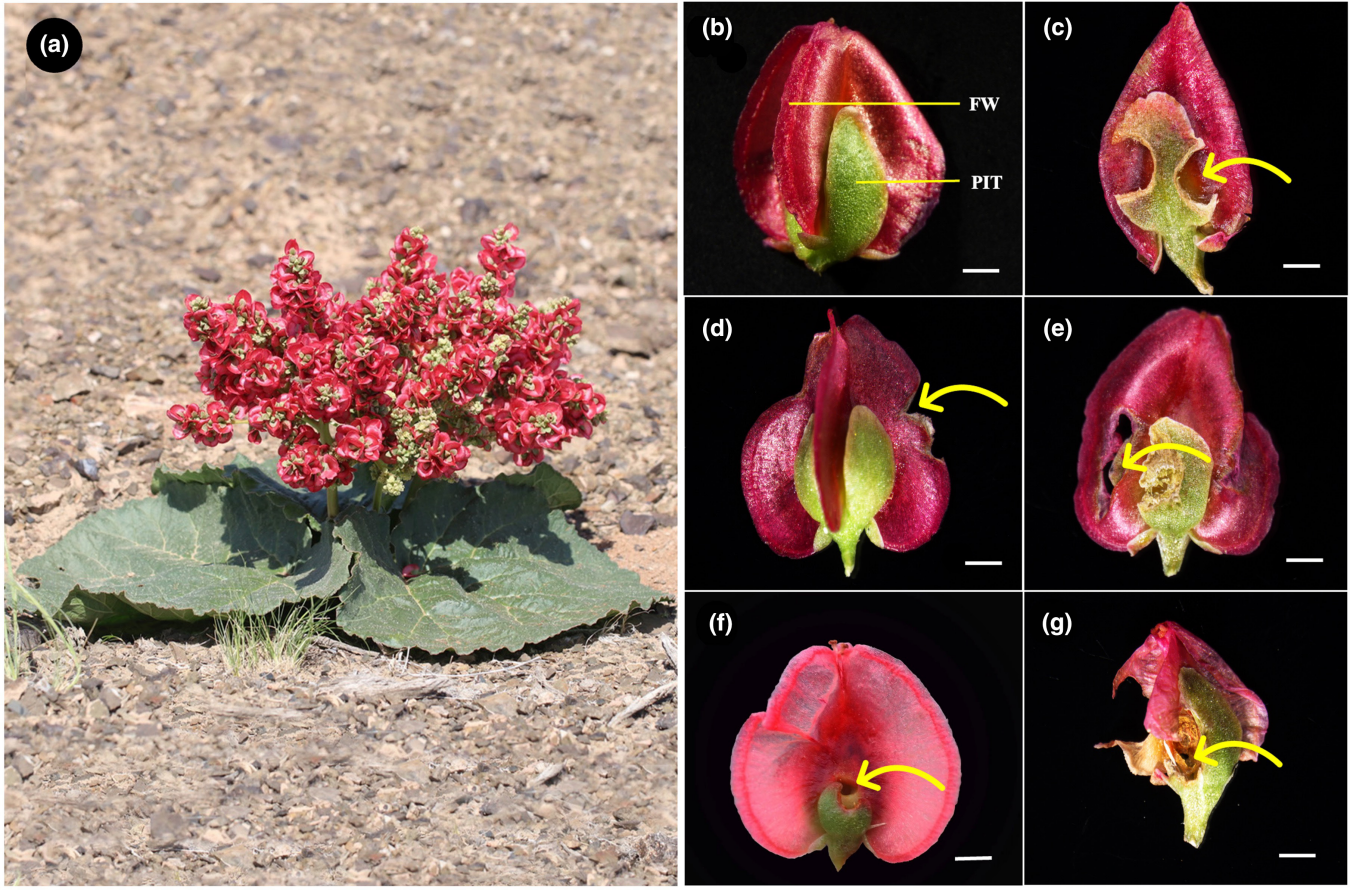
*Rheum nanum* diaspores representing different categories of insect herbivory. (a) The plant of *R*. *nanum*; (b) I (intact diaspores); (c) T (diaspores with damaged persistent inner tepals); (d) W (diaspores with damaged wings); (e) TW (diaspores with damaged both persistent inner tepals and wings); (f) TP (diaspores with damaged both persistent inner tepals and pericarp); (g) TWP (diaspores with damaged persistent inner tepals, wings, and pericarp); FW, fruit wing; PIT, persistent inner tepal. Scale bars = 2 mm. The arrow indicates where the diaspore was damaged by insects.

The study area is located in the Kalamaili Mountain Nature Reserve (88°30′–90°03′ E, 44°36′–46°00′ N) in the Junggar Basin of the Xinjiang Uygur Autonomous Region (hereafter Xinjiang), China. This area is an inland cold desert with gravelly desert soil and a temperate continental arid climate. Mean annual temperature is 3.8°C, and mean monthly temperatures of the coldest (January) and hottest (July) months are −19.3 and 22.8°C, respectively. Mean annual precipitation (including rain and snow) is 209.6 mm (National Meteorological Information Center, China Meteorological Data Network, http://data.cma.cn/), and annual potential evaporation is >2000 mm (Zheng et al., 2012). Each plant of *R*. *nanum* can produce 1–3 panicles from the tip of rhizome each year in the four investigated populations of study area, each panicle can produce 5–10 branches with more than 5 racemes, and there are more than 10 diaspores for each racemes (Figure 1a).

### 2.2 Insect herbivory of diaspores in natural populations

To determine the prevalence of insect herbivory in the four investigated populations of *R*. *nanum*, we randomly selected 30 plants with mature diaspores in each population in the Kalamaili Mountain Nature Reserve (Table 1) on 4 June 2022. We sampled all mature diaspores from each panicle at the upper, middle and lower branches of each plant (Table 1). Based on the part of the diaspore that was damaged by insects, we classified the diaspores from each plant into six categories (Figure 1): intact diaspores (I), diaspores with damaged persistent inner tepals (T), diaspores with damaged fruit wings (W), diaspores with both damaged persistent inner tepals and wings (TW), diaspores with both damaged persistent inner tepals and pericarps (TP), and diaspores with damaged persistent inner tepals, wings and pericarps (TWP). The percentage of diaspores with each category of insect herbivory was calculated as (Ni/Nt) × 100, where Ni is the number of diaspores with a particular category of insect herbivory and Nt is the total number of selected diaspores from each individual plant.

**TABLE 1:** Location of the collection sites of *Rheum nanum* diaspores in the Junggar Desert of Xinjiang, China.

Population	Collection site	Longitude (E)	Latitude (N)	Altitude (m a.s.l.)
P1	Fuyun County, Xinjiang, China	89°29′	45°29′	1062
P2	Fuyun County, Xinjiang, China	88°45′	45°16′	814
P3	Fuyun County, Xinjiang, China	89°24′	45°08′	958
P4	Jimsar County, Xinjiang, China	88°50′	44°56′	550

### 2.3 Seed abortion in relation to insect herbivory of diaspores

In the natural populations of *R*. *nanum*, the embryo in seeds is white and resilient, or sheetlike, almost transparent and inelastic. Results of TTC (2,3,5‐triphenyl tetrazolium chloride) test indicated that the white, resilient embryos were viable, but the sheetlike, almost transparent, inelastic embryos were not viable. In this study, we regarded seeds with a viable embryo as normal, and those with a non‐viable embryo or a completely predated/destroyed embryo as aborted.

In the four investigated populations of *R*. *nanum*, the P3 population had the most individuals and highest plant density. In order to collect enough diaspores for the demand of our experiment treats, we selected one raceme randomly at the upper, middle, and lower positions of more than 100 plants in P3 population on 5 June 2022, and collected all diaspores form these racemes. Furthermore, we put all these diaspores together and divided them into the six categories (I, T, W, TW, TP, TWP) of insect herbivory. Seeds and embryos were mechanically removed from diaspores, and embryo morphology of each seed was recorded. Four replicates of 25 diaspores for each of the six categories of insect herbivory were examined to determine the percentage of aborted seeds.

### 2.4 Effect of predispersal insect herbivory on seed development

#### 2.4.1 Mass of seeds and embryos

To determine the effect of predispersal herbivory on seed mass of *R*. *nanum*, we collected more than 200 matured and dried diaspores of I, T, W and TW from P3 population, and removed the seed from all diaspores. Then, each seed and embryo from 50 diaspores of I, T, W, and TW were weighed individually using an electronic balance (ME204E/02, Mettler Toledo Instruments (Shanghai) Co., LTD, China) with a precision of 0.0001 g. In TP and TWP, all seeds were aborted or eaten by insect larvae totally, we treated the mass of seeds and embryos as zero.

#### 2.4.2 Seed germination

To understand the seed germination differences in diaspores with the different categories of insect herbivory, the optimum temperature and light/dark conditions for germination of *R*. *nanum* seeds were tested firstly. We collected 1000 intact freshly matured diaspores from one raceme at the upper, middle, and lower positions of each of 100 plants in P3 population on 5 June 2022. Seeds were removed from these diaspores because the pericarp and wings of *R*. *nanum* have been shown to inhibit seed germination (unpublished data). Then, seeds were placed on two layers of Whatman No. 1 filter paper moistened with 2.5 mL of distilled water in 9‐cm‐diameter plastic Petri dishes. The Petri dishes were incubated in light (12‐h daily photoperiod) and constant darkness (Petri dishes with seeds in them wrapped with aluminum foil) at daily (12/12‐h) temperature regimes of 5/2°C, 15/2°C, 20/10°C, 25/15°C and 30/15°C. The 5/2°C regime represents February and December in the Junggar Basin of Xinjiang, China, 15/2°C March, early April and November, 20/10°C late April and October, 25/15°C May and September and 30/15°C June, July and August (Lu et al., 2010). Four replicates of 25 seeds were incubated for each treatment. The criterion for germination was emergence of radicle from seeds. Germination in light was examined daily for 28‐days, at which time germinated seeds were counted and removed from the Petri dishes; distilled water was added every day. To prevent light effects on seed germination at the dark conditions, we added an appropriate amount of distilled water to keep the filter paper moist in a green light every 7 days and counted the final germination percentage after 28‐day (Lu et al., 2020). After the germination trials were complete, TTC test was conducted on nongerminated seeds for viability to excluded the nonviable seeds from the calculations of germination percentages.

To determine the effect of predispersal insect herbivory on seed germination of diaspores with the different categories of insect herbivory, we collected matured diaspores with the six categories of insect herbivory from P3 population on 10 June 2021, and removed the seeds from I, T, W, and TW. Four replicates of 25 seeds for each of the four categories were incubated at optimum germination temperature regime (25/15°C) in light (12‐h daily photoperiod) for 28‐days, and the percentage of seed germination was determined. All seeds of TP and TWP were aborted, so we treated their germination percentage as zero.

### 2.5 Statistical analyses

All data were expressed as the mean ± SE. To meet the requirements of one‐way analysis of variance (ANOVA), all data were analyzed for normality and homogeneity of variance prior to analysis. If data were normal and homogeneous, they were subjected to further analysis. If data exhibited a non‐normal distribution or variances were not homogeneous, treatment differences were assessed by using the more conservative Kruskal–Wallis nonparametric test.

The Kruskal–Wallis nonparametric test was used to determine if there were differences in the percentage of diaspores with different categories of insect herbivory in natural populations, seed abortion in diaspores with different categories of insect herbivory and effect of predispersal insect herbivory on seed development.

A generalized linear model (GLM) with a logit‐link function with germination as a binomial response variable (two categories: germinated vs. nongerminated) was used to test the significance of the main effects (temperature and light) and their interaction on germination in “seed germination of freshly‐matured seeds” experiment in SPSS 26.0 (SPSS Inc., Chicago, IL, USA). The significance of the effects of temperature, light, and their interaction in the model was tested by Wald *χ* ^2^ values.

The least significant difference (LSD) test was performed for multiple comparisons to determine significant (*p* < .05) differences among categories of insect herbivory, in which the *p* value was adjusted by Bonferroni correction. All data analyses except those from the “seed germination of freshly‐matured seeds” experiment were performed with R 4.2.2 software (R Core Team, 2022).

## 3 RESULTS

### 3.1 Insect herbivory of diaspores in natural populations

In the four investigated *R*. *nanum* populations (Table 1), the percentage of diaspores damaged by insects was 22.83 ± 3.25% (P1), 17.49 ± 1.78% (P2), 14.37 ± 1.30% (P3), and 24.17 ± 1.94% (P4) (Table 2). In the diaspores with different categories of insect herbivory, percentage of I (intact diaspores) was significantly higher than that of the other five categories within the population (P1: H = 123.24, *df* = 5, *p* < .001; P2: H = 49.57, *df* = 5, *p* < .001; P3: H = 118.25, *df* = 5, *p* < .001; P4: H = 81.31, *df* = 5, *p* < .001). Percentages of T (diaspores with damaged tepals) and W (diaspores with damaged wings) were significantly higher than those of TP (diaspores with damaged both persistent inner tepals and pericarp) and TWP (diaspores with damaged persistent inner tepals, wings and pericarp) except in P3. However, percentage of T did not significantly differ from that of W (P1: *p* = 1.00; P2: *p* = .17; P3: *p* = .80; P4: *p* = .15), while the percentage of T was higher than that of TWP but not significantly (Table 2).

**TABLE 2:** Percentage (mean ± SE) of diaspores with different categories of insect herbivory in the four populations of *Rheum nanum*.

Population	Insect herbivory categories					
	I	T	W	TW	TP	TWP
P1	77.17 ± 3.25^a^	8.81 ± 1.94^b^	9.51 ± 1.23^b^	3.56 ± 1.31^bc^	0.35 ± 0.14^c^	0.24 ± 0.17^c^
P2	82.51 ± 1.78^a^	5.16 ± 0.87^bc^	9.07 ± 1.26^b^	2.57 ± 1.04^cd^	0.32 ± 0.20^d^	0.28 ± 0.20^d^
P3	85.63 ± 1.30^a^	3.71 ± 0.67^bc^	5.65 ± 0.67^b^	2.99 ± 0.48^bcd^	0.25 ± 0.10^d^	1.70 ± 0.37^cd^
P4	75.83 ± 1.94^a^	6.56 ± 0.65^bc^	10.63 ± 1.4^b^	4.64 ± 0.97^cd^	0.84 ± 0.28^d^	1.50 ± 0.29^d^

*Note*: I (intact diaspores); T (diaspores with damaged persistent inner tepals); W (diaspores with damaged wings); TW (diaspores with damaged both persistent inner tepals and wings); TP (diaspores with damaged both persistent inner tepals and pericarp); TWP (diaspores with damaged persistent inner tepals, wings, and pericarp). Different lowercase letters indicate significant differences among different insect herbivory categories (*p* < .05).

### 3.2 Seed abortion in relation to insect herbivory of diaspores

The fate of seeds differed among the six categories of insect herbivory in the four investigated populations of *R*. *nanum*. In I, T, W, and TW, most seeds had intact endosperm and viable embryos, but some seeds in these four categories had a non‐viable embryo. In TP, part of the endosperm had been eaten by insect larvae, and all embryos were nonviable. In TWP, insect larvae had eaten all of the endosperm and embryo, leaving an empty pericarp.

**FIGURE 2:**
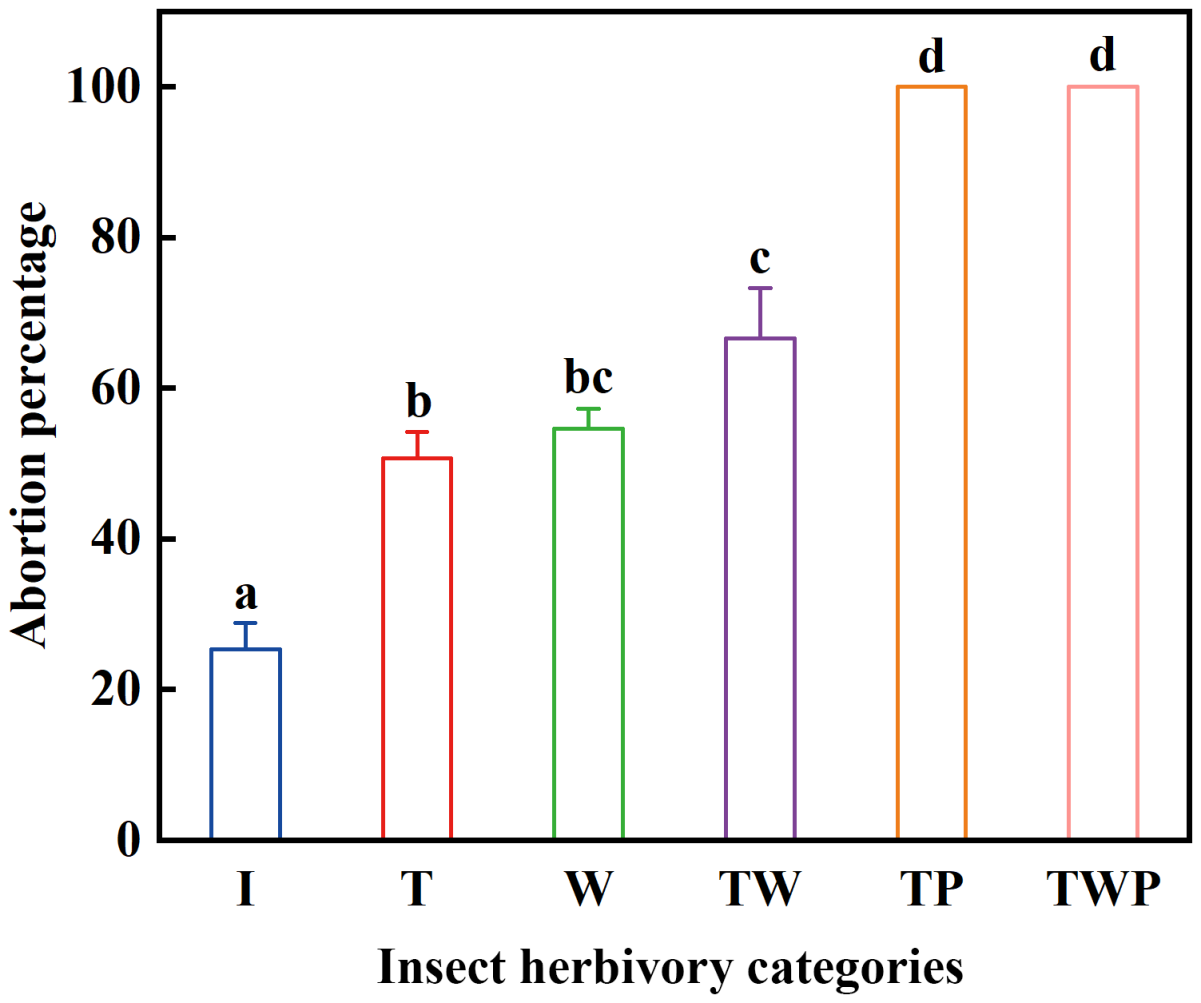
Seed abortion percentage (mean ± SE) of *Rheum nanum* diaspores with different categories of insect herbivory. I (intact diaspores); T (diaspores with damaged persistent inner tepals); W (diaspores with damaged wings); TW (diaspores with damaged both persistent inner tepals and wings); TP (diaspores with damaged both persistent inner tepals and pericarp); TWP (diaspores with damaged persistent inner tepals, wings and pericarp). Different lowercase letters indicate significant differences among different categories of insect herbivory (*p* < .05).

The seed abortion percentage of I was significantly lower than that of the other five categories of insect herbivory (T, W, TW, TP, and TWP) (all *p* < .05) (Figure 2). The percentages of seed abortion for T, W, and TW were significantly lower than those for TP and TWP (all *p* < .05), and all seeds of TP and TWP were aborted (Figure 2).

### 3.3 Effect of predispersal insect herbivory on seed development

#### 3.3.1 Mass of seeds and embryos

**FIGURE 3:**
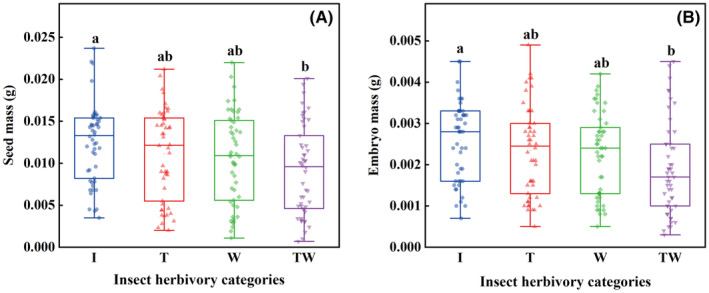
Mass (mean ± SE) of seeds (A) and embryos (B) from *Rheum nanum* diaspores with different categories of insect herbivory. I (intact diaspores); T (diaspores with damaged persistent inner tepals); W (diaspores with damaged wings); TW (diaspores with damaged both persistent inner tepals and wings). Different lowercase letters indicate significant differences among different insect herbivory categories (*p* < .05).

Predispersal insect herbivory significantly influenced mass of seeds (H = 8.66, *df* = 3, *p* < .05) and of embryos (H = 12.09, *df* = 3, *p* < .01) in *R*. *nanum*. There were no significant differences in seed mass among I, T, and W (all *p* > .05), whereas seed mass of TW was significantly lower than that of I (*p* < .05). Embryo mass showed the same trend as seed mass (Figure 3). All embryos of TP and TWP were aborted.

#### 3.3.2 Seed germination

**FIGURE 4:**
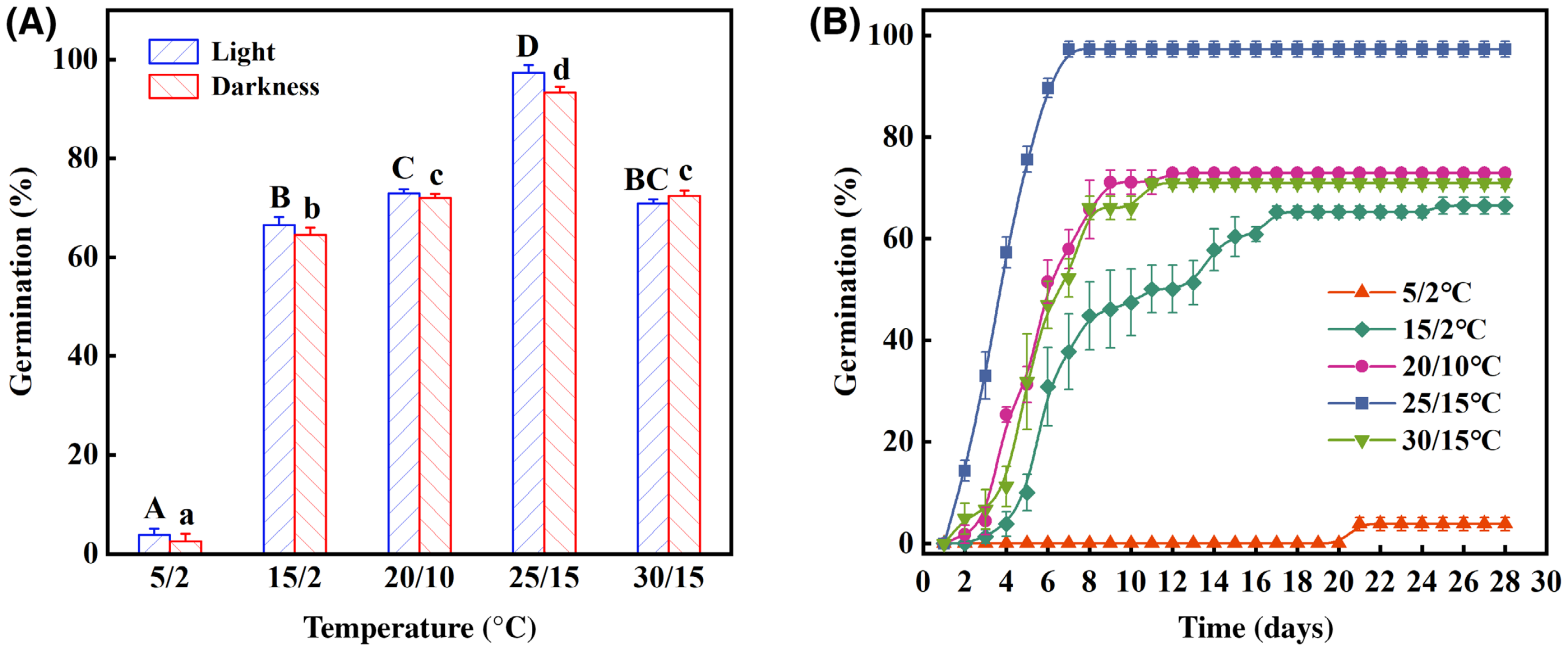
Final germination percentages (mean ± SE) of freshly‐matured *Rheum nanum* seeds in light and darkness (A) and cumulative germination percentages (mean ± SE) of freshly matured seeds in light (B). Different uppercase letters indicate significant differences among different temperature regimes in light and different lowercase letters significant differences among different temperature regimes in darkness (*p* < .05).

GLM analysis indicated that the germination percentage of intact freshly matured seeds of *R*. *nanum* was significantly affected by temperature (*χ* ^2^ = 112.81, *df* = 4, *p* < .001) but not affected by light (*χ* ^2^ = 0.58, *df* = 1, *p* = .45) and the interaction between temperature and light (*χ* ^2^ = 0.66, *df* = 4, *p* = .96) (Table 3). Final germination of freshly matured seeds was >90% at 25/15°C in both light and darkness (Figure 4A). Maximum germination of freshly matured seeds was 97.3% at 25/15°C after incubation in light for 7‐days (Figure 4B) and 93.3% in darkness at 25/15°C for 28‐days (Figure 4A), which was significantly higher than that of the other four temperature regimes (all *p* < .05). Consequently, the optimum temperature regime for germination of freshly matured seeds of *R*. *nanum* in light was 25/15°C.

**TABLE 3:** GLM of the effects of light, temperature and their interaction on germination of freshly matured seeds of *Rheum nanum*.

Factors	*df*	Wald‐*χ* ^2^	*p* Value
Light (L)	1	0.583	.445
Temperature (T)	4	112.805	<.001
L × T	4	0.660	.956

**FIGURE 5:**
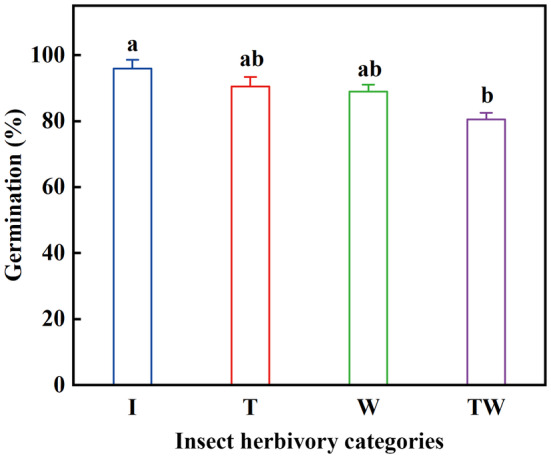
Final germination percentage (mean ± SE) of *Rheum nanum* seeds excised from diaspores with different categories of insect herbivory and incubated at the optimum temperature regime (25/15°C) in light. I (intact diaspores); T (diaspores with damaged persistent inner tepals); W (diaspores with damaged wings); TW (diaspores with damaged both persistent inner tepals and wings). Different lowercase letters indicate significant differences among different categories of insect herbivory (*p* < .05).

Predispersal insect herbivory had a significant effect on seed germination (*H* = 9.85, *df* = 3, *p* < .05). Under optimum temperature conditions, seed germination percentages of T and W did not differ from that of I (all *p* > .05), but germination of TW was significantly lower than that of I (*p* < .05) (Figure 5). No seeds of TP and TWP germinated due to aborted embryos.

## 4 DISCUSSION

Although the protective effects of thorns, spines, and trichomes on plants have been previously reported (Bitew, 2018; Kariyat et al., 2017; Lev‐Yadun, 2001; Xing et al., 2017), there is limited information regarding the role of fruit appendages in protecting seeds during development. We found that persistent inner tepals and fruit wings confer mechanical protection against predispersal insect herbivory of developing fruits and seeds. This finding sheds light on the importance of fruit appendages in ensuring the successful development and survival of seeds.

### 4.1 Prevalence of predispersal insect herbivory

In the cold desert of the Junggar Basin of Xinjiang, China, the percentage of *R*. *nanum* diaspores damaged by insects ranged from 14% to 24% across four populations (Table 2). Similarly, Han et al. (2018) observed that seed predation of *Astragalus lehmannianus* (Fabaceae) was 16%–22% in the cold desert of northern Xinjiang. However, in the foothills of Wenatchee Mountains Chelan County, Washington (USA), seed predation of another Fabaceae species, *A*. *sinuatus*, was 65%–82% (Combs et al., 2011). The reason for these differences in seed predation among different habitats can be attributed to the environmental conditions under which the mother plants are grown such as resource availability, plant density, predator abundance, and plant defenses (Fröborg & Eriksson, 2003; Moreira et al., 2016; Xu et al., 2015). Moreover, in *R*. *nanum*, the percentage of diaspores with damaged pericarps was lower than that of diaspores with damaged persistent inner tepals and/or fruit wings (Table 2). This results suggests that the persistent inner tepals and fruit wings are more exposed and susceptible to be damaged by insects.

### 4.2 Seed abortion in relation to insect herbivory of diaspores

Fruit appendages have a protective function not only postdispersal, but also predispersal. For example, the fruit spines of *Tribulus cistoides* (Zygophyllaceae) can protect the mature mericarp from post‐dispersal seed predation by Darwin's finches (Johnson et al., 2020), the trichomes in *Lachnoloma lehmannii* (Brassicaceae) fruits protect seeds against loss of viability from overheating on a hot sandy soil surface after seed dispersal (Mamut et al., 2014). Meanwhile, fruit wings of *Zygophyllum potaninii*, *Z*. *lehmannianum*, and *Z*. *macropterum* (Zygophyllaceae) can provide predispersal protection for seeds against insect herbivory (Xie et al., 2023).

In our study, the seed abortion percentages of *R*. *nanum* diaspores with different categories of insect herbivory were significantly higher than those of intact diaspores. Seeds of all diaspores with a damaged pericarp were aborted (Figure 2), because such injury directly results in the abortion of embryos (El‐Atta, 1993). Furthermore, seed abortion percentages of *R*. *nanum* diaspores with a damaged persistent inner tepals and/or fruit wings were significantly lower than those of diaspores with a damaged pericarp (Figure 2). These results suggest that both persistent inner tepals and fruit wings can act as a barrier, which can reduce the impact of predispersal insect herbivory on seed development by decreasing the damage to the pericarp, endosperm and embryo (Bao & Grabovskaya‐Borodina, 2003). Similar mechanical protection of the mature seed also has been observed in some species of *Pisum*, *Vigna* (Fabaceae) (Howe & Currie, 1964), and *Lithocarpus* (Fagaceae) (Chen et al., 2012). The hard outer seed (or fruit) coat of these species could prevent insect larvae from entering seed, thereby avoiding injury to the embryo.

### 4.3 Effect of predispersal insect herbivory on seed development

In our investigation of *R*. *nanum*, seed mass did not decrease significantly when persistent inner tepals or fruit wings were damaged respectively (Figure 3A), suggesting that insect herbivory on persistent inner tepals or fruit wings had no effect on seed mass. Additionally, the seed mass of diaspores with damaged persistent inner tepals and fruit wings significantly decreased (Figure 3A), indicating that insect herbivory to both persistent inner tepals and fruit wings had negative effect on seed mass. The same trend was observed for embryo mass (Figure 3B). However, all seeds of TP (diaspores with both damaged persistent inner tepals and pericarps) and TWP (diaspores with damaged persistent inner tepals, wings and pericarps) were aborted. These results suggest that the fruit wings and persistent inner tepals alleviate the effect of insect herbivory on seed development by reducing insect herbivory on the seed and thereby protecting the seeds. Interestingly, in the case of *Elettaria cardamomum* (Zingiberaceae), persistent bracts had no significant effect in providing resistance against thrips (Jacob et al., 2020). This highlights the variation in the protective functions of different fruit appendages across plant species.

At 15/25°C, the germination percentage of fresh seeds of *R*. *nanum* was over 90% within four weeks (Figure 4A), and over 90% of fresh seeds germinated within 7 day in light (Figure 4B). These indicate that *R*. *namum* seeds are nondormant (Baskin & Baskin, 2014) and this result were also reported in other 17 species of Polygonaceae (Zhang, Willis, et al., 2021). Furthermore, temperature significantly affected seed germination, but light conditions did not (Table 3). These results reveal that light is not a limiting factor for seed germination in *R*. *nanum*, and temperature and water conditions in the desert might be crucial factors (Zhang, Hu, et al., 2021). The nondormant feature of *R*. *nanum* seeds allows itself to germinate at different light conditions when soil moisture and temperature are suitable in desert environments.

Predispersal insect herbivory not only affects fruit development, but it also may affect seed germination (Han et al., 2018). Compared to intact diaspores of *R*. *nanum*, all seeds from TP (diaspores with damaged persistent inner tepals and pericarp) and from TWP (diaspores with damaged persistent inner tepals, wings, and pericarp) were aborted. However, damage to persistent inner tepals or fruit wings had no effect on seed germination (Figure 5). These results indicate that persistent inner tepals and fruit wings ensure seed germination via reducing insect damage to seeds (Wang et al., 2019). Moreover, damage to both the persistent inner tepals and fruit wings had a negative effect on seed germination (Figure 5). A possible explanation is that insect herbivory on both persistent inner tepals and fruit wings significantly affects seed development, leading to a significant decrease seed germination. Consistent with our findings, a previous study on three *Zygophyllum* (Zygophyllaceae) species also demonstrated that their fruit wings can provide protection against insect herbivory during fruit development, thus reducing the effects on seed development and seed germination (Xie et al., 2023). Consequently, the persistent inner tepals and wings of the *R*. *nanum* provide protection for the seeds to alleviate harm from insect predation predispersal, and thus improving the fitness of the seeds and ensuring the survival and reproduction of this species.

## 5 CONCLUSION

In conclusion, we have demonstrated that persistent inner tepals and fruit wings of *R*. *nanum* act as a barrier and provide mechanical protection for its seed to alleviate the damage from insect herbivory predispersal. As a result, *R*. *nanum* has more likelihood of successful reproduction under the harsh environment conditions of Central Asian cold deserts. These findings may provide new insights into the function of fruit appendages of desert plants. Therefore, in order to advance our knowledge of adaptive evolution of fruits with appendages in desert plants, more investigation about the ecological role of fruit appendages is required.

## AUTHOR CONTRIBUTIONS

**Yuting Li:** Data curation (lead); investigation (lead); methodology (equal); writing – original draft (lead); writing – review and editing (equal). **Jannathan Mamut:** Conceptualization (supporting); methodology (equal); writing – review and editing (equal). **Kaiqing Xie:** Investigation (supporting); methodology (supporting). **Jing Zhao:** Data curation (supporting). **Dunyan Tan:** Conceptualization (lead); funding acquisition (lead); supervision (lead); writing – review and editing (equal).

## FUNDING INFORMATION

The National Natural Science Foundation of China, Grant/Award Number: 32071668; The Natural Science Foundation of Xinjiang Uygur Autonomous Region of China, Grant/Award Number: 2022D01E49; The Grant of Innovation Environment Construction of the Xinjiang Uygur Autonomous Region, China, Grant/Award Number: PT2315. Grant for Wild Plant & Animal Resources Survey of Urumqi City from Xinjiang Forestry and Grass Bureau of China.

## CONFLICT OF INTEREST STATEMENT

The authors declare no conflict of interest.

## Data Availability

The data that support the findings in the present study are available at: https://datadryad.org/stash/share/1KJJIvw5ZrDCSaJt1Xa_D‐6M0fZynliFNYFt1NY6vuE.
